# Valve-sparing root replacement with short saphenous vein interposition for left coronary artery reconstruction in redo type A aortic dissection: a case report

**DOI:** 10.1093/jscr/rjag243

**Published:** 2026-04-09

**Authors:** Ryoji Kinoshita, Taiju Watanabe, Kazuya Okaguchi, Kazunobu Hirooka

**Affiliations:** Department of Cardiovascular Surgery, Tsuchiura Kyodo General Hospital, 4-1-1 Otsuno, Tsuchiura, Ibaraki 300-0028, Japan; Department of Cardiovascular Surgery, Tsuchiura Kyodo General Hospital, 4-1-1 Otsuno, Tsuchiura, Ibaraki 300-0028, Japan; Department of Cardiovascular Surgery, Tsuchiura Kyodo General Hospital, 4-1-1 Otsuno, Tsuchiura, Ibaraki 300-0028, Japan; Department of Cardiovascular Surgery, Tsuchiura Kyodo General Hospital, 4-1-1 Otsuno, Tsuchiura, Ibaraki 300-0028, Japan

**Keywords:** redo aortic surgery, aortic dissection, valve-sparing root replacement, saphenous vein graft, coronary reconstruction, bailout strategy

## Abstract

Redo aortic root surgery for acute type A aortic dissection is technically demanding, particularly when coronary reconstruction is complicated by tissue fragility. Reliable bailout strategies are required when direct coronary reimplantation is unsafe during valve-sparing root replacement. A 52-year-old man previously underwent total arch replacement with a frozen elephant trunk for acute type A aortic dissection. He later developed progressive aortic root dilatation and a proximal anastomotic pseudoaneurysm, and redo valve-sparing root replacement was performed. Intraoperatively, the left coronary artery was severely dissected and unsuitable for reimplantation. A short segment saphenous vein graft was interposed between the trimmed left main trunk and the neo-aortic root. Postoperative imaging confirmed preserved valve function and satisfactory coronary flow. Short-segment saphenous vein interposition represents a practical bailout option in complex redo aortic root surgery.

## Introduction

Redo aortic root replacement after previous surgery for Stanford type A aortic dissection remains technically demanding, particularly when coronary complications arise. Failure of coronary reimplantation is a recognized cause of perioperative morbidity and mortality. Non-direct coronary reimplantation or unplanned coronary bypass during redo root surgery has been associated with increased operative risk [1, 2]. The optimal bailout strategy in redo valve-sparing root replacement with fragile dissected coronary tissue remains unclear. We report a case in which a short segment saphenous vein graft (SVG) was used for left coronary reconstruction when direct reimplantation proved unsafe.

## Case presentation

**Figure 1:**
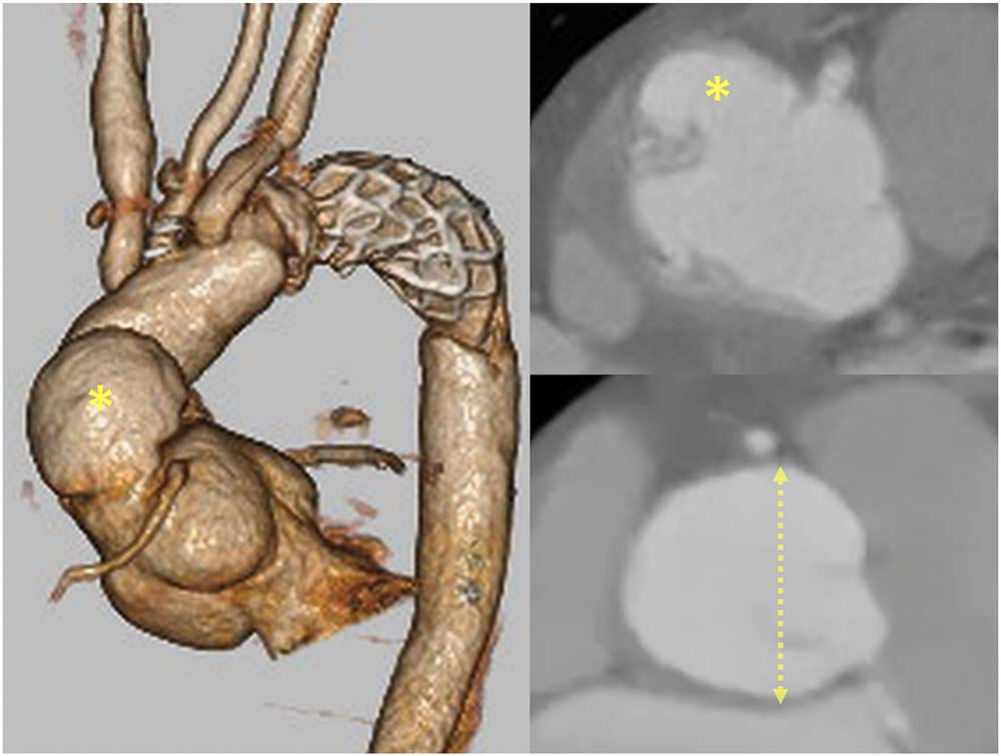
Preoperative CT showing the pseudoaneurysm (asterisk) and aortic root dilation (dotted line; 46 mm).

A 52-year-old man underwent emergency total arch replacement with a frozen elephant trunk for acute type A aortic dissection 1 year prior. Aortic root dilation (43 mm) and severe regurgitation were present at the initial event, but a supra-coronary replacement was selected. Follow-up imaging revealed enlargement of the root to 46 mm and a 37 × 23 mm proximal pseudoaneurysm (Fig. 1).

Redo valve-sparing root replacement using the remodeling technique with a 26-mm Valsalva graft was performed. Dense adhesions were encountered. After mobilization of the coronary buttons, the left coronary artery (LCA) was reimplanted in standard fashion. However, test perfusion revealed severe bleeding due to unrecognized dissection with adventitial delamination (Video 1).

**Figure 2:**
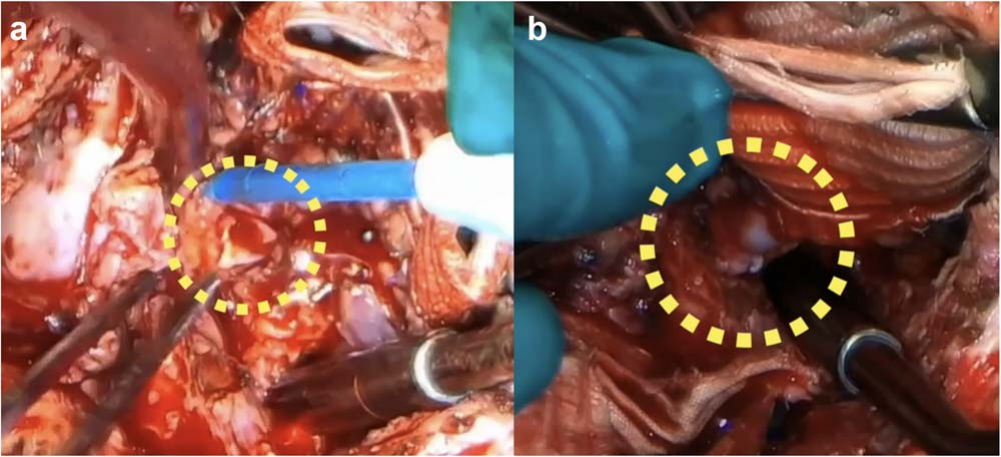
Intraoperative finding: (a) The LCA was trimmed to the left main trunk due to dissection and fragility. (b) Final reconstruction with a short SVG interposition.

Reconstruction using a 7-mm prosthetic graft interposition with bovine pericardial reinforcement was attempted. Persistent bleeding from the fragile coronary wall prevented secure hemostasis. The button was therefore trimmed to a healthy left main trunk (Fig. 2a), creating a gap unsuitable for direct reimplantation. A 1-cm segment of saphenous vein graft was harvested and interposed between the left main trunk and the neo-root (Fig. 2b, Video 2). The right coronary artery was reimplanted conventionally.

**Figure 3:**
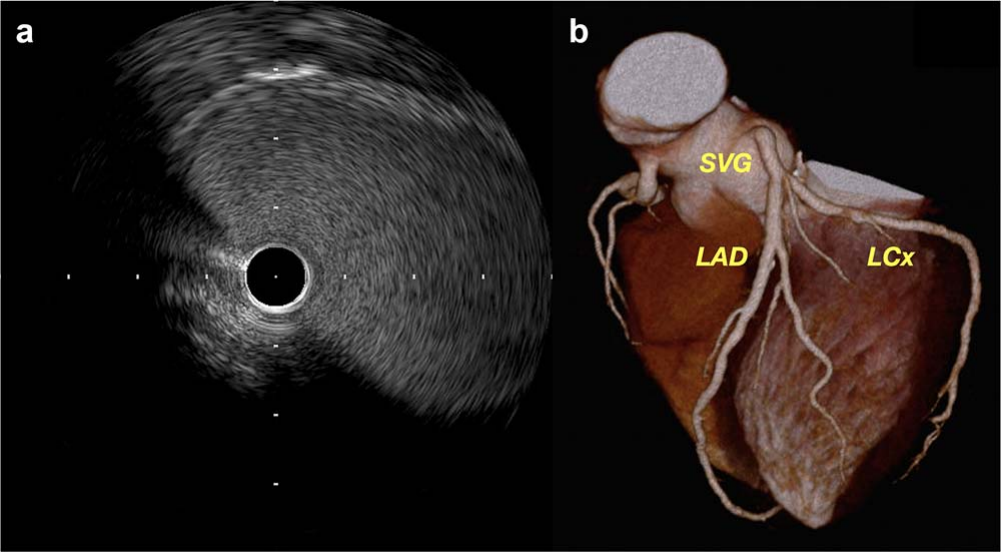
(a) Postoperative IVUS demonstrating a widely patent SVG with no stenosis. (b) Follow-up coronary CT at 6 months showing excellent graft configuration.

Recovery was uneventful. Coronary angiography and intravascular ultrasound (IVUS) on postoperative day 20 demonstrated graft patency without flow disturbance (Fig. 3a, Video 3). Fractional flow reserve was 0.88. Six-month computed tomography (CT) confirmed sustained patency (Fig. 3b, Video 4).

## Discussion

Coronary reconstruction is a key determinant of success in redo root surgery. Non-standard coronary reconstruction has been associated with increased mortality [3].

### Carrel reimplantation

The Carrel button technique remains the gold standard because it preserves physiologic coronary flow and geometry [4]. However, adequate Valsalva wall and coronary tissue integrity are required. In this case, severe dissection and adventitial delamination made secure reimplantation impossible.

### Prosthetic interposition

Piehler and modified Cabrol techniques allow tension-free coronary reattachment, with acceptable early and midterm outcomes [5, 6]. However, these methods require structurally reliable coronary tissue and secure hemostasis. Despite trimming to the left main trunk and reinforcement, persistent bleeding occurred with prosthetic interposition. Given intrinsic tissue fragility, prosthetic reconstruction was considered unsafe due to the risk of uncontrolled bleeding or late pseudoaneurysm.

### CABG-only strategy

 Coronary artery bypass grafting (CABG) has been used in advanced coronary malperfusion in acute dissection [7, 8]. However, it abandons direct aortic–coronary continuity. In this case, the left main trunk remained salvageable, global ischemia was absent, and durable antegrade inflow was preferred. CABG-only was therefore considered a theoretical option rather than a definitive solution.

### Biological interposition

Superficial femoral artery interposition has been reported for coronary reconstruction [9, 10]. Arterial conduits may offer superior durability; however, conduit selection must consider anatomical compatibility. In this patient, trimming resulted in a 4–5 mm left main trunk. In a middle-aged male, superficial femoral artery diameter would likely be 7–8 mm, creating size mismatch and technical difficulty.

Saphenous vein grafts provide configurational flexibility and can be tailored to match coronary diameter and orientation. Kazui *et al.* reported successful saphenous vein interposition in complex root pathology [11]. Based on anatomical considerations, a short-segment saphenous vein graft was selected.

The graft was kept short (~1 cm) and straight to minimize kinking and geometric distortion. Native coronary continuity was preserved without ligation. Mild curvature was observed postoperatively; however, no flow limitation or pressure gradient was detected. Given the dynamic aortic root environment, long-term imaging surveillance has been planned.

### Postoperative medical management

Because saphenous vein grafts are susceptible to early thrombosis and intimal hyperplasia, dual antiplatelet therapy was initiated. Evidence from coronary bypass surgery supports improved early venous graft patency with dual antiplatelet therapy [12]. Statins were continued for their vascular protective effects, including modulation of smooth muscle proliferation and inflammation [13]. Colchicine was administered to suppress postoperative vascular inflammation, although evidence for graft durability remains evolving.

Long-term durability of short-segment saphenous vein interposition in the aortic root position remains uncertain; therefore, strict surveillance and optimized medical therapy are essential.

## Conclusion

When direct coronary reimplantation is precluded by fragile dissected tissue in redo aortic root surgery, short-segment saphenous vein interposition represents a pragmatic and adaptable bailout option. Careful conduit selection, attention to graft geometry, and comprehensive postoperative management are critical to support long-term graft patency.

## Supplementary Material

Video_1_rjag243

Video_2_rjag243

Video_3_rjag243

Video_4_rjag243

Supplementary_Video_caption_rjag243

## Data Availability

Data sharing is not applicable to this article as no datasets were generated or analyzed during the current study.
