# Supplementary material for: Valve-sparing root replacement with short saphenous vein interposition for left coronary artery reconstruction in redo type A aortic dissection: a case report
Source: J Surg Case Rep. 2026 Apr 9;2026(4):rjag243. doi: 10.1093/jscr/rjag243 (PMC13070380; doi:10.1093/jscr/rjag243)
Supplement: Supplementary_Video_caption_rjag243 [file supplementary_video_caption_rjag243.docx]

Video 1: Intraoperative finding of remodeling VSRR and active bleeding from the dissected LCA during the initial test perfusion.

Video 2: Final surgical view showing the aortic root and the interposed SVG.

Video 3: Postoperative coronary angiography confirming graft patency.

Video 4: Three-dimensional reconstruction of the 6-month follow-up CT.
